# Endoscopic iliopsoas lengthening for treatment of recalcitrant iliopsoas tendinitis after total hip arthroplasty

**DOI:** 10.1093/jhps/hnac052

**Published:** 2023-03-07

**Authors:** John C Bonano, Kinsley Pierre, Christopher Jamero, Nicole A Segovia, James I Huddleston, Marc R Safran

**Affiliations:** Department of Orthopaedic Surgery, Stanford Hospitals and Clinics, 450 Broadway Street, Redwood City, CA 94063, USA; Department of Orthopaedic Surgery, Stanford Hospitals and Clinics, 450 Broadway Street, Redwood City, CA 94063, USA; Department of Orthopaedic Surgery, Stanford Hospitals and Clinics, 450 Broadway Street, Redwood City, CA 94063, USA; Department of Orthopaedic Surgery, Stanford Hospitals and Clinics, 450 Broadway Street, Redwood City, CA 94063, USA; Department of Orthopaedic Surgery, Stanford Hospitals and Clinics, 450 Broadway Street, Redwood City, CA 94063, USA; Department of Orthopaedic Surgery, Stanford Hospitals and Clinics, 450 Broadway Street, Redwood City, CA 94063, USA

## Abstract

Iliopsoas (IP) tendinitis from impingement upon the acetabular component after total hip arthroplasty (THA) has been treated with open and endoscopic IP tenotomy or acetabular component revision. This study describes the results of a consecutive series of patients treated with endoscopic IP tenotomy as a less invasive alternative. Twenty-eight patients with IP impingement after THA underwent endoscopic IP lengthening from 2012 to 2021 at a single-center academic institution. The follow-up of 24 of these patients was achieved with a mean follow-up of 7.6 months (range 1–28). Outcomes included the modified Harris Hip Score (mHHS), visual analog pain scale (VAS), satisfaction, component positioning and complications. Seventy-one percent of patients were satisfied or very satisfied after their operation. The median mHHS preoperatively was 57 (Interquartile range [IQR] 43–60) and postoperatively was 75 (IQR 66–92, *P* < 0.001). Clinically meaningful improvements in mHHS were seen in patients with VAS pain scores <5, cup prominence >8 mm, body mass index >30, and less than 2 years from their index THA. Two patients developed a deep infection 7 and 10 months postoperatively (neither related to the release), and one patient underwent open psoas release for persistent impingement. Endoscopic IP tenotomy is a safe and effective treatment for impingement after THA. Patients with cup prominence >8 mm, body mass index >30 and less than 2 years since their index THA may have more clinically meaningful improvements in pain and function.

## INTRODUCTION

Iliopsoas (IP) tendinitis after total hip arthroplasty (THA), felt to be a result of IP impingement upon the acetabular component, causes persistent groin pain in up to 4.3% of patients after an otherwise successful THA [1, 2]. Symptoms are often aggravated by active hip flexion or passive hip extension [3, 4], which causes tendon irritation as it impinges against the anterior edge of the acetabular cup when proud of the anterior acetabular wall or femoral head [1, 5, 6]. IP tendinitis has been correlated with insufficient cup anteversion and cup protrusion beyond the anterior edge of the acetabulum, which may contribute to impingement [7]. Other causes include irritation from cement fragments, long screws or osteophytes [1, 5, 6].

Conservative treatment options for IP tendinitis following hip arthroplasty include rest, ice, anti-inflammatory medications and physical therapy. Corticosteroid injections may provide symptomatic relief as well as diagnostic information. Symptom resolution with nonoperative treatment can be expected in only 47–50% of patients [8, 9]. Operative treatment is indicated after the failure of nonoperative measures. Historically, surgical options included either open IP tenotomy and/or acetabular component revision [6, 10–12]. However, IP lengthening through endoscopic (tenotomy at the lesser trochanter) or arthroscopic (tenotomy off the acetabular rim) techniques has recently gained popularity since they are much less invasive when compared with the other alternatives of open IP lengthening or acetabular component revision [6, 12]. Potential benefits of endoscopic tenotomy include easier visualization of the tendon insertion on the lesser trochanter, avoiding potential intra-articular damage to the acetabular and femoral components and reduced risk of nerve injury. Several small case series have reported initial success using both of these techniques [9, 13–22]. Within these studies, six used a transcapsular arthroscopic technique [9, 13–17], three used an endoscopic release at the level of the lesser trochanter [19, 21, 22] and one used both endoscopic and arthroscopic techniques [20]. A variety of clinical outcomes were reported among these studies including patient-reported outcome measures, groin pain resolution and complications.

Given the variability in surgical techniques and outcomes, the efficacy of endoscopic IP lengthening is not clearly understood. The purpose of this study is to report the results of a consecutive series of patients treated with endoscopic IP tenotomy for IP tendinitis after THA and to identify factors that may be associated with the success of this procedure. Our primary outcome was the modified Harris Hip Score (mHHS). Secondary outcomes included the International Hip Outcome Tool 12 (iHOT-12), visual analog pain scale (VAS), patient satisfaction, graded psoas strength and complications. Radiographs were reviewed to determine acetabular component positioning.

## MATERIALS AND METHODS

### Patient and demographic data

**Fig. 1.**
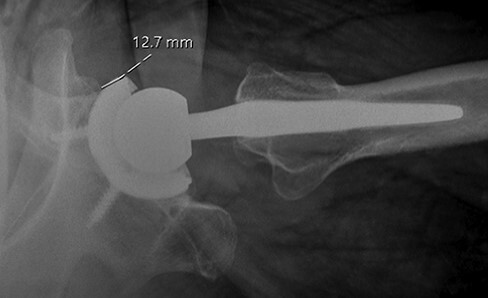
Cross-table lateral radiograph of the right hip measuring the amount of acetabular component prominence from the anterior acetabular rim.

A consecutive series of 28 patients with IP tendinitis after THA were treated with endoscopic IP lengthening by a single surgeon (M.R.S.) from 2012 to 2021 at a single-center academic institution. We used Current Procedural Terminology code 29999 to identify patients who underwent endoscopic IP lengthening and then excluded patients who did not have a prior ipsilateral THA at the time of their surgery. Institutional review board approval was obtained, and all patients provided written consent to participate in the study. Patient demographic and clinical data included age, gender, body mass index (BMI), affected side and date of index THA. All patients completed a mHHS at their initial preoperative visit. Patient history was reviewed to characterize symptom duration, pain location and aggravating factors. Clinical examination included graded hip flexion strength, and all patients obtained anterior-posterior (AP) pelvis and cross-table lateral radiographs preoperatively. Additional workup and treatment measures were obtained at the surgeon’s discretion based on this clinical assessment and included magnetic resonance imaging, erythrocyte sedimentation rate (ESR) or C-reactive protein (CRP) and ultrasound or computerized tomography-guided injection of local anesthetic and/or corticosteroid in the psoas tendon sheath performed by a radiologist or physiatrist. Radiographs were reviewed to determine acetabular component positioning. Cup prominence was determined on a cross-table lateral radiograph by measuring the amount of acetabular component overhang from the anterior acetabular rim (Fig. 1) [2, 6, 9]. Acetabular component anteversion was also measured on the cross-table lateral radiograph using the method described by Woo and Morrey as the angle formed when a tangential line to the opening of the acetabulum and a line drawn perpendicular to the horizontal edge of the radiograph intersect [23]. Inclination was measured on the AP pelvis radiograph by drawing a line through the ischial tuberosities and a line tangential to the face of the acetabular cup.

### Endoscopic IP tenotomy surgical technique

**Fig. 2.**
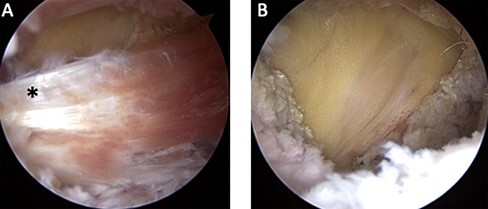
Intraoperative images showing (A) the iliopsoas tendon (asterisk) at its insertion on the lesser trochanter and (B) post-tenotomy retraction of the iliopsoas tendon.

Indications for surgery included persistent groin pain despite nonoperative treatment in patients with IP tendinitis after a prior THA. Endoscopic IP tenotomy was performed by a single surgeon with 25 years of hip arthroscopy experience. The patient was positioned supine on a standard fracture table. The operative leg was flexed 20° and then externally rotated under fluoroscopic guidance until the lesser trochanter was visually maximized. Traction was not applied to the extremity. Two portals were established—a distal anterolateral and an IP portal, as described by Ilizaliturri *et al.* [24]. The IP tendon was identified just proximal to its insertion onto the lesser trochanter. Hook electrocautery was used to transect the tendon just proximal to its bony insertion, with subsequent tendon retraction indicating complete release (Fig. 2). Care was taken not to cut the muscular attachment. An anterior capsular release was not performed. All surgeries were performed as outpatient surgeries in a surgery center. No patients were admitted or had an overnight stay. All patients were provided with the same postoperative instructions, which included protected weight-bearing until walking without a limp and no resisted hip flexion of 6 weeks.

### Outcome measures

Patients were followed in the senior author’s clinic and were also emailed four surveys to complete: (i) VAS, (ii) mHHS, (iii) five-point patient satisfaction scale and (iv) iHOT-12. Patient charts were reviewed to evaluate for postoperative complications, including readmission, dislocation, infection and reoperation.

### Statistical analysis

A Wilcoxon signed-rank test was used to analyze the differences in pre- and postoperative mHHS. Mann–Whitney tests were used to compare predictive variables for differences in patient satisfaction and to analyze the change in mHHS among categorically grouped radiographic, demographic and outcome measures. Analyses were completed in RStudio version 1.1.456 (Boston, MA) using a two-sided level of significance of 0.05. Clinically significant improvements in mHHS were determined using an established minimal clinically important difference of 9.0 at a mean of 6 months following surgery [25].

## RESULTS

Twenty-eight patients were identified who underwent endoscopic IP lengthening following THA for recalcitrant IP tendinitis between 2012 and 2021 at a single-center academic institution. Twenty-four patients, 8 men and 16 women, were available for follow-up at a mean of 7.6 months (range 1–28 months, Table I). The average age at surgery was 69 years (IQR 63–74). The mean time from THA to endoscopic surgery was 2.7 years (IQR 1.5–6). All patients localized pain to their groin and had significant temporary relief from at least one image-guided IP injection with local anesthetic. Twenty of those patients (83%) had preoperative corticosteroid injections as well. ESR and CRP were obtained for six patients, and advanced imaging was obtained for six patients. No patients underwent a hip aspiration.

**Table I.** Demographics and preoperative variables of patients who underwent endoscopic iliopsoas tenotomy for impingement after total hip arthroplasty

*Variable*	*Value*
Age^a^	69 (62.8–74.3)
Body mass index^a^	30.4 (25.2–34.3)
ASA^a^	2 (2–3)
Male (%)	8 (33%)
Years since THA^a^	2.7 (1.5–6)
Steroid injection	20 (83%)
Advanced imaging	6 (25%)
Cup prominence (mm)^a^	7.0 (2.3–12)
Cup inclination (degrees)^a^	45 (37.8–49)
Cup version (degrees)^a^	30 (24.5–38)

a Data reported as a median (IQR).

ASA = American Society of Anesthesiologists Classification.

The median mHHS preoperatively was 57 (IQR 43–60) and postoperatively was 75 (IQR 66–92, *P* < 0.001, Table II). Larger improvements in mHHS were associated with increased patient satisfaction (*P* = 0.005) and lower VAS pain scores (*P* = 0.030). The median acetabular component inclination, anteversion and prominence for patients in our series were 42.5°, 31.5° and 5.75 mm, respectively. Acetabular component position did not have a statistically significant impact on mHHS, but cup prominence of >8 mm was associated with clinically meaningful improvements (Table III). Patients with a BMI of >30 who had their index THA within 2 years also had clinically meaningful improvements in their postoperative mHHS. The median postoperative iHOT-12 score was 71 (IQR 48–80). There was no significant change in graded psoas strength (*P* = 0.15). At final follow-up, 71% of patients were either very satisfied or satisfied with their surgery and satisfaction was associated with improvement in mHHS (*P* = 0.005) and shorter time from index THA (*P* = 0.084, Table IV).

**Table II.** Outcome scores and complications after endoscopic iliopsoas tenotomy

*Variable*	*IP tenotomy (n = 24)*	*P value*
Preoperative mHHS^a^	57 (43–60)	<0.001
Postoperative mHHS^a^	75 (66–92)	
iHOT-12^a^	71 (48–80)	
VAS current (1–10)^a^	3 (0.8–5)	
Satisfaction^b^		
Very satisfied	12 (50%)	
Satisfied	5 (21%)	
Neutral	2 (8%)	
Dissatisfied	4 (17%)	
Very dissatisfied	1 (4%)	

a Data reported as a median (IQR).

b Satisfaction was measured on a 5-point scale where 1 indicates very satisfied and 5 very dissatisfied. Data are presented as total number of patients.

VAS = visual analog scale for pain.

**Table III.** Patient pre-/postoperative mHHS and predictive variables after endoscopic iliopsoas tenotomy

		*Pre-mHHS*		*Post-mHHS*		*Difference*		
		*Median*	*IQR*	*Median*	*IQR*	*Median*	*IQR*	*P value*
Prominence	<8 mm	58.8	47.8–66.8	76.0	64.5–96	14.6	3.9–35.7	0.154
	>8 mm	48.4	38.2–57.1	75.0	69–83.5	27.2^a^	24.5–37.4	
Index THA	>2 years	45.1	39.0–57.7	68.0	62.8–79.5	14.7	10.3–33.3	0.494
	<2 years	57.1	51.6–62.6	82.0	71–98	26.9^a^	22.0–37.0	
BMI	<30	57.1	53.3–61.0	69.0	64.5–81.5	14.5	6.0–27.2	0.072
	>30	47.3	40.7–57.1	85.0	69–96	32.7^a^	18.3–40.9	
Satisfaction	Satisfied	57.1	45.1–60.4	84.0	74–96	31.9^a^	22.0–40.9	0.005
	Dissatisfied	49.5	41.7–58.2	61.5	59–63	3.3	0.4–14.5	
VAS pain	<5	57.1	46.2–59.9	82.0	70–96	26.9^a^	14.6–39.9	0.030
	>5	41.7	40.7–58.2	61.5	59–63	3.3	0.4–18.3	

a A clinically meaningful improvement in mHHS based on a minimal clinical important difference of 9.0 at a mean of 6 months.

VAS = visual analog scale for pain.

**Table IV.** Patient satisfaction and predictive variables after endoscopic iliopsoas tenotomy

	*Very satisfied + satisfied (n = 17)*		*Very dissatisfied + dissatisfied (n = 5)*		
	*Median*	*IQR*	*Median*	*IQR*	*P value*
Prominence	10	3–12	3.9	0–4.3	0.113
Inclination	45	38–50	40	39–40	0.182
Version	31.5	26.5–39	25.7	19–38	0.321
Pre-mHHS	57.1	45.1–60.4	49.5	41.7–58.2	0.695
Post-mHHS	84	74–96	61.5	59–63	< 0.001
mHHS difference	31.9	22–40.9	3.3	0.4–14.5	0.005
Body mass index	33	29.2–34.5	26.3	25–31.3	0.196
Years since index THA	5	1.5–7	2	1.4–2	0.084

Two patients developed infections at 7 and 10 months postoperatively, requiring a two-stage THA revision. One of these patients had previously undergone a revision for aseptic loosening of the femoral stem 1 year prior to endoscopic psoas lengthening. In both cases, the infection was not felt to be related to the endoscopic procedure. One patient underwent an open psoas release for persistent impingement. This patient had persistent, yet mild, pain 5 years after the open procedure. There were no admissions, 90-day readmissions or episodes of postoperative instability at the final follow-up.

## DISCUSSION

The purpose of this study was to describe the demographics and outcomes of a consecutive series of patients treated with this minimally invasive technique at our institution from 2012 to 2021 and to identify factors that may be associated with the success of this procedure. In the present series, all but one patient had improvements between their pre- and postoperative mHHS. Statistically significant improvements in mHHS were found to be associated with increased patient satisfaction and lower VAS pain scores. We also found that patients with a BMI >30, a cup prominence of >8 mm and patients who had their index THA within 2 years of their IP tenotomy achieved clinically meaningful improvements in mHHS.

To date, there are only small case series evaluating arthroscopic/endoscopic IP tenotomy for IP tendinitis due to IP impingement after THA [9, 13–22]. Only a few studies have specifically evaluated an endoscopic, extra-articular technique involving release of the IP tendon at the level of the lesser trochanter. Gedouin *et al.* [21] reported on a series of 10 patients and found that 8 had complete symptom relief and were very satisfied. Bell *et al.* [19] reported on a series of 60 patients, and 93.3% had resolution of their pain. They found that BMI and increased offset were associated with IP symptoms after THA. The French Arthroscopy Society performed a prospective multicenter endoscopic/arthroscopic iliopsoas lengthening study in patients with IP impingement after THA [20]. Sixty-four patients underwent either endoscopic or arthroscopic IP lengthening (only 30% as outpatient surgery) and reported alleviation of pain in 92% and 87% patient satisfaction; however, 11% reported pain with passive stretching of the IP and 21% with pain with seated hip flexion [20]. However, none of these studies reported on potential prognostic factors for the success of endoscopic tenotomy. Viamont-Guerra *et al.* [22] reported a single-center series of 50 patients undergoing IP lengthening after THA attempting to identify risk factors for surgical failure. While 8% ultimately underwent revision hip arthroplasty, the authors reported that 91% had achieved the patient-acceptable symptomatic state (PASS) using the Oxford Hip Score; however, only 57% reached the PASS threshold when assessed with the mHHS. Furthermore, while the authors noted clinically important improvements in the mHHS in 76% and 89% of patients using the Oxford Hip Score, they noted persistent groin pain in 43% of patients (including moderate pain in 11%). Those authors were unable to identify risk factors, though they acknowledged that the analysis was difficult to measure precisely using CT. In our series, we found that the timing of the tenotomy within 2 years of THA may be related to treatment success. One potential explanation for this finding is that early treatment may prevent the development of chronic tendinopathic changes, including neovascularization and mucoid degeneration, that may cause persistent pain postoperatively [26].

In this series, the acetabular component position did not have a statistically significant impact on mHHS, but cup prominence of >8 mm was associated with more clinically meaningful improvements. While anteversion and inclination have implications on THA stability [27], IP irritation occurs when the anterior edge of the cup extends beyond the native bony acetabulum, and thus cup prominence is the radiographic factor most predictive of IP impingement. Lower cup anteversion [28] and a larger difference in anteversion between the native acetabulum and the prosthetic acetabular component [29] have been shown to result in increased anterior cup protrusion. Cyteval *et al.* [30] found that acetabular cup overhang of more than 12 mm was sensitive for diagnosing symptomatic IP impingement, while cup overhang of less than 8 mm was not associated with impingement. Chalmers *et al.* [9] reported in their series that there was a higher rate of treatment success for patients with a cup prominence of >8 mm if they underwent acetabular component revision rather than a psoas tenotomy. The authors reported that in their psoas tenotomy treatment group, groin pain resolved in only 1/3 patients with cup prominence >8 mm and in 5/5 patients with <8 mm of cup prominence. In contrast, in our series, we found that there was a clinically significant improvement in mHHS for those patients with a cup prominence of >8 mm, indicating that these patients can potentially obtain symptom relief without the need for open revision surgery.

In our series, all patients were treated with an endoscopic tenotomy without any postoperative complications. This is similar to the studies by Gedouin [21] and Bell [19], where there was only one minor complication involving a postoperative hematoma that resolved with conservative treatment, while Guichard noted two complications—one hip dislocation and one postoperative hematoma. In contrast, complication rates following open tenotomy and acetabular component revision have been cited as high as 33.3% and 19.4%, respectively [10, 11]. One patient in our series underwent an open psoas release for persistent impingement, and one patient did not have any improvement in their mHHS. It is unclear why these patients did not improve postoperatively. Both patients had relief from a preoperative steroid injection, and complete release of the tendon at the level of the lesser trochanter was confirmed in all cases. It may be related to cup design/position, muscular tightness/imbalance, posture or capsular tethering. The cup prominence for both patients was <8 mm, which we found was associated with less mHHS improvement. We did not perform a capsular release so further studies are needed to help determine its role in the treatment of IP tendinitis after THA.

There are several limitations to this retrospective review. We acknowledge that our sample size was small and may be subjected to selection bias given that patients were treated by a single surgeon. We also recognize that our follow-up period may not be long enough to exclude the recurrence of symptoms. We did not have access to the majority of the patients’ THA operative reports, as their THAs were generally performed at outside institutions, and therefore, we were unable to determine component sizes and the type of approach used. We also did not have preoperative CT scans, which could have provided a more accurate assessment of cup prominence. Nonetheless, many studies have used plain radiographs to determine component position in the way that was described in this study [2, 6, 9].

## CONCLUSION

In conclusion, this case series supports the use of endoscopic IP tenotomy as a safe alternative to open tenotomy or acetabular component revision for symptomatic IP tendinitis after THA, presumably due to impingement against the acetabular component. Patients can expect high satisfaction and significant improvements in pain and function with low complication rates. Furthermore, we found that patients with a cup prominence of >8 mm who were treated within 2 years of their index THA had clinically meaningful improvements in their mHHS.

## DATA AVAIILABILITY

The authors confirm that all data generated or analyzed during this study are included in this published article. Raw data that support the findings of the study are available from the corresponding author, upon reasonable request.
